# Opportunities and Challenges for Gut Microbiota in Acute Leukemia

**DOI:** 10.3389/fonc.2021.692951

**Published:** 2021-07-07

**Authors:** Tao Ma, Yan Chen, Li-Juan Li, Lian-Sheng Zhang

**Affiliations:** ^1^ Department of Hematology, Lanzhou University Second Hospital, Lanzhou, China; ^2^ Department of Hematology, The Affiliated Hospital of Southwest Medical University, Luzhou, China

**Keywords:** gut microbiota, acute leukemia, pathogenesis, GVHD, drugs, immunity, allo-HSCT

## Abstract

Acute leukemia (AL) is a highly heterogeneous hematologic malignancy, and although great progress has been made in the treatment of AL with allogeneic hematopoietic stem cell transplantation (Allo-HSCT) and new targeted drugs, problems such as infection and GVHD in AL treatment are still serious. How to reduce the incidence of AL, improve its prognosis and reduce the side effects of treatment is a crucial issue. The gut microbiota plays an important role in regulating disease progression, pathogen colonization, and immune responses. This article reviews recent advances in the gut microbiota and AL pathogenesis, infection, treatment and its role in allo-HSCT.

## Introduction

Acute leukemia (AL), a kind of hematologic malignancy, mainly includes acute myeloid leukemia (AML) and acute lymphoblastic leukemia (ALL) (1). In 2018, a global survey of 36 cancers in 185 countries indicated that leukemia ranks fifteenth in cancer incidence and tenth in cancer deaths (2). The most common cancer in children is AL (3). More than 80% of children with ALL are long term survivors, compared with 20% to 40% of adults (4). In AML, the five-year survival rate is 40% for patients younger than 60, and only 10-20% for patients older than 60 (5). Cytotoxic chemotherapy, ionizing radiation, genetic mutations, benzene, and some blood system diseases (such as myelodysplastic syndrome, paroxysmal nocturnal hemoglobinuria) can lead to AL (6, 7). The specific pathogenesis of AL is not fully understood, and some types of AL are dangerous, ineffective and have a high mortality rate. Immune system plays an important role in the pathogenesis, progression and treatment of AL. AML patients have higher T regulatory cells (Tregs) and CD8^+^ T cells compared to normal donors (8). However, AML patients have T-cell dysfunction, which is associated with immune escape of the disease (9). Moreover, Tregs are a major contributor to defective immune responses (10). In AML patients, increased Tregs can suppress effector T cells, and the more Tregs, the worse the treatment effect (11). Treatment with chimeric antigen receptor T-cell therapy is highly effective in patients with ALL, which is also associated with the immune system (12).

There are four major colonization sites of microorganisms in the human body: oral, vagina, skin, and gut (13). Gut microbiota colonize the newborn following birth (14). The gut microbiota is composed of native flora and transient flora in food intake (15). It is estimated that each individual’s gut microbiome includes more than 100 different species (15). Gut microbiota has recently been recognized as an important factor in regulating disease progression, pathogen colonization, and immune responses (1, 13, 16), and the pathogenesis, progression, and treatment of AL have been linked to the immune system. Gut microbiota has also been linked to diabetes, Alzheimer’s disease, tumor and so on (17–19). This review summarizes the progress of the interaction between gut microbiota and AL.

## The Role of Gut Microbiota in the Pathogenesis of AL

The pathogenesis of AL is unclear. Greaves M propose a model for ALL, in which some children are born with pre-leukemic clones, but acquired infections cause key genetic mutations that develop into ALL (20). Some studies have found that newborns carry leukemia-related mutations, such as TCF3-PBX1 and ETV6-RUNX1 (21, 22). Exposure to infection promotes the occurrence of ALL in newborns carrying these genes (23, 24). In mice with PAX5 or ETV6-RUNX1, ALL was caused by a disruption in the microbiome caused by antibiotic treatment early in life, and the gut microbiome of PAX5-carrying mice differed from the wild type (25). A study enrolled 81 subjects, including 58 ALL patients and 23 healthy controls, using 16S rRNA quantitative arrays and bioinformatics analysis to examine their stool specimens (26). Many species of bacteria appeared significantly different in stool specimens from ALL patients compared to healthy controls, such as a significant enrichment of *Bacteroides clarus* in ALL (26). Whether gut microbiota disturbance can cause AL in humans needs further study.

Alterations in some gut microbiota species and metabolites may promote the development of leukemia. In a mouse model of leukemia, the proportion of bacteria with the function of converting dietary flavonoids in the small intestine of leukemic mice was increased (27). It has been suggested that maternal intake of bioflavonoids may lead to infant and young child leukemia (28). A national case-control study conducted in Denmark and found that infections of the gastrointestinal tract were associated with an increased risk of AML (29). So why does the occurrence of a gastrointestinal infection lead to an increased risk of developing AML? We assume that the imbalance of gut microbiota due to gastrointestinal infections causes immune disorders that lead to AML, but more studies are still needed to verify this.

The risk of AL increased significantly during childhood or adolescence after the cumulative dose of ionizing radiation (30). Ionizing radiation is the accepted cause of leukemia. A study found that mice that survived high doses of radiation had unique gut microbiota, with Lachnospiraceae and Enterococcaceae as the most abundant bacteria (31). Then, they collected stool samples from 21 leukemia patients who had received total body radiation before hematopoietic stem cell transplantation, patients with shorter duration of diarrhea (diarrhea less than 10 days) had more Lachnospiraceae and Enterococcaceae in their feces than patients with longer duration of diarrhea (diarrhea more than 10 days) (31). Lactobacillaceae also increased in patients with shorter duration of diarrhea, but there was no statistical significance (31). Bone marrow and small intestinal epithelial cells are highly sensitive to ionizing radiation, and they are the main site of radiation injury (32). Short-chain fatty acids (SCFAs), a metabolite of gut microbiota, reduces DNA damage and reactive oxygen species release in blood and gastrointestinal tissues during radiation damage (31). Fecal microbiota transplantation (FMT) improved the survival rate of irradiated animals, increased white blood cells, and improved gastrointestinal function (33). The gut microbiota plays a key role in host defenses against radiation, as well as protecting the hematopoietic and gastrointestinal systems.

Benzene is a widespread air pollutant and a component of gasoline, industrial emissions, automobile exhaust and tobacco smoke (34). Traffic-related benzene exposure can cause acute myeloid leukemia in children (35). A study of benzene exposure in mice showed that benzene exposure altered the gut microbiota, with increased level of Actinobacteria, and Helicobacter were observed in the stools of mice exposed to high doses of benzene, and increased level of Actinobacteria were significantly and negatively correlated with basic blood cells (white blood cell, red blood cell, and hemoglobin levels) (36). Whether gut microbiota plays a role in benzene causing leukemia remains to be studied.

It is well known that naïve helper T cells (Th0) can be transformed into Th1, Th2, Treg and Th17 cells etc. Th1 produces the cytokine interferon gamma (IFN-γ) that exerts anti-tumorigenic effects in the tumor microenvironment, while Th2/Treg produces the cytokines interleukin 4 (IL-4), IL-5, IL-10, etc. that mediate pro-tumorigenic effects (17). In some solid tumor models, depletion of the gut microbiota leaded to a significant increase in IFN-γ-producing T cells and a decrease in IL-17A and IL-10-producing T cells, thereby reduced tumor burden. However, this phenomenon was not present in mice lacking mature T and B cells, suggesting that the action of the gut microbiota on tumor cells required the active involvement of adaptive immunity (17). In innate and adaptive immunity, microbiota plays an important role in its maturation, development and functions (37).

There is no doubt that the development of AL is related to the dysregulation of the human immune system, and the gut microbiota also regulates the human immune system, which has been shown to be closely related to the development of pancreatic cancer, colon cancer and other tumors (38, 39). Whether the gut microbiota is also involved in the development of AL by modulating human immunity needs to be studied in more depth.

## A Normal, Diverse Gut Microbiota Is Critical to Human Immunity and Can Predict Infection in Patients With AL

Infection is a common symptom in patients with newly diagnosed AL. In addition, the bone marrow suppression that occurs after chemotherapy in AL patients is also prone to infection. Infection is an important cause of death in patients with AL (40, 41). Gut microbiota is strongly associated with host immune function and resistance to infection. It is essential for the host to maintain a healthy immune system (42). It has been shown that gut microbiota can produce inhibitory products to suppress vancomycin-resistant enterococci (43). The use of carbapenem antibiotics in the empirical treatment of fever due to neutropenia in patients with AL reduces gut microbiota diversity and increases colonization by vancomycin-resistant enterococci (44). SCFAs, a metabolite of gut microbiota, promote the differentiation of T lymphocytes into effector T cells and regulatory T cells by inhibiting histone deacetylase (45). SCFAs also significantly inhibited the activity and infectivity of E. coli and protected intestinal epithelial cells from damage caused by C. difficile toxins (45, 46). Treatment of mice with broad-spectrum antibiotics resulted in a decrease in the number of hematopoietic stem cells and pluripotent progenitor cells in their bone marrow, as well as a decrease in granulocytes and B cells in the bone marrow and an increase in CD8+ T cells (47). This is the result of broad-spectrum antibiotics interfering with the STAT1 signaling pathway by consuming the gut microbiota (47). The aromatic amino acid metabolite production pathway of intestinal gut symbiont Clostridium sporogenes produces 12 compounds, 9 of which accumulate in host serum, indolepropionic acid being one of them. A decrease in indolepropionic acid increases the number of immune cells in mice, including neutrophils, monocytes, and memory T cells, and mice with decreased indolepropionic acid have greater intestinal permeability and are more likely to trigger an inflammatory response in mice (48). A normal and diverse gut microbiota is essential for human immunity and resistance to infection.

Causes of gut microbiota dysbiosis in AL patients associated with antibiotic use, nutrition, and chemotherapy (49). A study used a 16s rRNA-based analysis of bacterial flora in oral and fecal specimens from 97 AML patients every two weeks from induction chemotherapy to neutrophil recovery. They found that fecal flora diversity and high levels of Porphyromonadaceae at the time of induction chemotherapy were positively associated with a low risk of infection (50). In AML patients who developed infection during induction chemotherapy, baseline fecal flora diversity was significantly lower than in patients who did not develop infection, and flora diversity gradually decreased over the course of induction chemotherapy (51). In a study of ALL, chemotherapy was also found to have an impact on the diversity of the gut microbiota, with a significant decrease in the relative abundance of certain bacterial groups (e.g., Bacteroidetes) and a significant increase in the relative abundance of other groups (e.g., Clostridiaceae and Streptococcaceae) following chemotherapy (52). In contrast to the findings of some studies (44), this study concluded that antibiotic use does not alter the diversity of the gut microbiota (52). However, it has also been suggested that vancomycin use significantly reduces gut microbial diversity in patients with ALL, while no correlation was found with piperacillin tazobactam, antifungal drugs and other antibiotics (53). Some investigators concluded that the composition, but not the diversity, of the gut microbiome prior to chemotherapy was an independent predictor of febrile neutropenia during chemotherapy. The predominance of Aspergillus phylum and Enterococcus (relative abundance ≥30%) predicted febrile neutropenia; Enterococcus (relative abundance ≥30%) and Streptococcaceae predominate in predicting diarrheal disease (52). Intestinal nutrition deficiency can lead to loss of intestinal epithelial barrier function and increased susceptibility to infection (54).

In a study evaluating the effect of probiotics on chemotherapy-induced gastrointestinal side effects in patients with AL, patients with AL were randomized to an oral Lactobacillus rhamnosus group and a control group. The primary endpoint was the incidence of gastrointestinal side effects, with significant reductions in nausea, vomiting, and abdominal distention (P<0.05) and a relative risk of diarrhea (0.5) in the oral probiotics group (55). Patients in the control group of this study did not take placebo and subjective error may occur. In a study of gut microbiota recovery in mice and humans, it was found that neither probiotics nor spontaneous recovery restored gut microbiota diversity after 4 weeks of disruption cessation with antibiotics. Importantly, probiotics significantly delayed recovery to baseline microbiome richness at all time points tested, compared to spontaneous recovery (56). However, gut microbiota diversity was indistinguishable to control after 8 days of fecal transplantation (56). The use of probiotics can only restore one or a few specific gut microbes, but not the function of a complex microbial community. Therefore, supplementation with one probiotic alone may have adverse effects on the host (49), or may even result in probiotic-induced infections. A patient who received autologous hematopoietic stem cell transplantation took probiotic-rich yogurt due to severe diarrhea, and a week later the patient developed infectious shock (57). Strain-specific PCR analysis showed that the pathogen was Lactobacillus rhamnosus GG, which was consistent with the probiotics in the yogurt (57). Another child with Philadelphia chromosome-positive ALL underwent chemotherapy and developed neutropenic fever with blood cultures suggestive of Bifidobacterium breve, which may be pathogenic when host immunity is reduced (58). In patients with AL, the diversity and composition of gut microbes correlate with whether or not an infection occurs or what type of infection occurs, and they are influenced by factors such as chemotherapy and antibiotics. Probiotics can reduce gastrointestinal symptoms such as diarrhea, but opportunistic infections with probiotics have also been reported in the presence of neutrophil deficiency.

The gut microbiota is closely related to the normal immunity of the organism (42). Among the infections present in patients with AL, the gut microbiota has been associated with diarrhea, neutropenic fever, and sepsis, and its diversity and composition are predictive of infection in patients (45–52, 59).

## Gut Microbiota Affects a Wide Range of Drugs Used to Treat AL

There is increasing evidence that host responses to chemotherapeutic agents can be influenced by the gut microbiota, primarily in terms of promoting drug efficacy; abrogation of anticancer effects; and modulation of toxicity (60). Gut microbiota regulates chemotherapeutic drugs through key mechanisms such as reduced diversity, ecological variation, translocation, immune regulation, metabolism, enzymatic degradation (60).

Cyclophosphamide (CTX) is commonly used to treat ALL, lymphoma, and other tumors. Viaud S et al. investigates the role of gut microbiota in CTX therapy through a mouse model (61). Non-myeloablative doses of CTX and adriamycin treated mice resulted in shortened small intestinal villi, disrupted epithelial barrier, and interstitial edema 48 hours after drug application (61). After the disruption of the intestinal barrier, some intestinal microorganisms are transferred to the mesenteric lymph nodes and spleen, and intestinal microorganisms including Lactobacillus johnsonii, Lactobacillus murinus and Enterococcus hirae can be cultured in these lymphoid organs (61). CTX treatment induces selective translocation of gut microbiota, for example, leading to reduced abundance of Lactobacillus and Enterococcus in the small intestine of mice. Some intestinal bacteria (Lactobacillus johnsonii, Lactobacillus murinus and Enterococcus hirae) stimulate the production of specific “pathogenic” T helper 17 (pT(H)17) cells. Killing these bacteria with antibiotics reduced the pT(H)17 response in tumor-bearing mice and made them resistant to CTX. Adoptive transplantation of pT(H)17 cells could recover the anti-neoplastic efficacy of CTX in part. In another study, calorie restriction was found to significantly enhance the gut microbiota with Lactobacillus and Trichinella, which are shown to relieve inflammation and improve intestinal barrier function. Calorie restriction reshapes stronger gut microbiota and may help reduce side effects of CTX (62).

Methotrexate (MTX) has the function of anticancer and immunosuppressive, and is one of the main drugs used in the treatment of ALL, central nervous system leukemia, and rheumatic immune system diseases (63–65). Common side effects of MTX include mucositis and intestinal damage (66, 67). MTX has significant effects on the amounts, diversity and major components of the mouse gut microbiota (68). MTX -treated mice showed reduced intestinal microbial diversity and changes in the composition of the gut microbiota, such as a dramatic decrease in the number of ruminal cocci and a dramatic increase in the number of members of the Lachnospiraceae (68). B. fragilis was significantly reduced by MTX treatment, and there was a tendency for the proliferation rate of B. fragilis to decrease with increasing M1 macrophage density (68). The mucosal muscle lesions seen in mtx-treated mice were significantly relieved by gavage with fragile B (68). Fragile B. markedly attenuated the MTX-induced increase in M1 macrophages, and the decrease in fragile B. in turn aggravated the MTX-induced macrophage imbalance, resulting in positive feedback, which led to intestinal tissue injury (68). Chronic long-term MTX exposure induced changes in the gut microbiota composition and function, with the relative abundance of the Firmicutes being higher than that of the Bacteroidetes at low doses of MTX, but the opposite at high doses of MTX. The relative abundance of the Firmicutes was positively correlated with the 48-h fecal excretion of 2,4-diamino-N-10-methylpteroic acid (DAMPA), a metabolite of MTX. Long-term exposure to MTX can alter the composition and function of the microbiome, which in turn affects its ability to detoxify MTX (69). MTX largely alters the human gut microbiota. Changes in gut flora and gene abundance were different between patients who responded to MTX and those who did not. MTX affects a variety of conserved human gut bacterial pathways, leading to reduced host immune activation (70).

PD-1 is a kind of immune checkpoint, PD-1 binding with PD-L1 will inhibit T cells and block the anti-tumor immune response. Anti-PD1 has been approved by FDA for the treatment of melanoma, non-small cell lung cancer, renal cell carcinoma and other tumors (71). In a mouse model, enhanced PD-1 expression on circulating CD8 T cells leads to the progression of AML (72). A phase II clinical trial of azacitidine combined with nivolumab in patients with relapsed/refractory AML obtained an encouraging result, with an overall response rate (ORR) of 33% (73). PD-1 inhibitors have promising applications in the treatment of AL, and the gut microbiota also interacts with PD-1 inhibitors. In a mouse model study of epithelial tumors, primary resistance of tumors to immune checkpoint inhibitors (ICIs) was found to be attributable to unusual composition of the gut microbiome, and the clinical benefit of ICIs in patients with late-stage cancer could be inhibited by antibiotics (74). Germ-free or antibiotic-treated mice receiving fecal microbiota transplantation (FMT) from patients with tumors that responded to ICIs improved the antitumor effects of PD-1 blockade, but FMT from patients with tumors that did not respond to ICIs had no such effect (74). Metagenomics of patient fecal samples revealed a correlation between the relative abundance of Akkermansia muciniphila and patient response to ICIs. Administration of oral supplementation of A. muciniphila to mice unresponsive to FMT restored the efficacy of PD-1 blockade in an interleukin-12-dependent manner (74). In another study of melanoma patients, researchers found that the effect of anti-pd-1 immunotherapy was associated with greater abundance of Bifidobacterium longum, Collinsella aerofaciens, and Enterococcus faecium in the patients’ stools (75). Transplanting feces from patients who responded to anti-pd-1 into germ-free tumor-bearing mice enhanced the T-cell effect and improved tumor control (75). In a study of the gut microbiota of melanoma patients receiving PD-1 immunotherapy, investigators found that the diversity of the flora and the relative abundance of Ruminococcaceae family were significantly higher in patients who responded to PD-1 immunotherapy (76). In immunotherapy for liver cancer, researchers also found more taxa richness and higher gene counts in the stools of patients who responded to anti-PD-1 (77). Gut microbiota correlates with the efficacy of ICIs in a wide range of tumors, with higher diversity of gut microbes associated with better efficacy. Whether the efficacy of anti-PD-1 therapy in patients with AL is also associated with the gut microbiota needs to be urgently investigated.

There are no studies on the interaction of gut microbiota with drugs used to treat AML (e.g., erythromycin, cytarabine, etc.), which is a future direction to investigate the relevance of gut microbiota to AL therapeutic drugs.

## The Place of Gut Microbiota in Allo-HSCT: for Better or for Worse?

Allo-HSCT is currently one of the most effective treatments for AL (78). Allo-HSCT means that hematopoietic stem cells collected from a healthy donor are infused into a patient with leukemia, and the donor’s immune cells can attack the leukemia cells to produce a graft-versus-leukemia (GVL) effect (79). The main side effect of allo-HSCT is graft-versus-host disease (GVHD) (79, 80). GVHD means that the immune cells of the donor attack the normal tissues of the patient, and the common organs are the skin, liver, and gastrointestinal tract, and the patient may present with diarrhea, vomiting, abdominal pain, jaundice, sclerosis of the skin, and other harmful manifestations (79). Acute graft-versus-host disease (aGVHD) occurs in 30-50% of all allo-HSCT patients, and chronic graft-versus-host disease (cGVHD) occurs in 35-50% of patients (81, 82). Prevention of GVHD includes the use of cyclosporine, tacrolimus, mycophenolate mofetil (MMF), methotrexate, etc (83). Despite the use of these drugs, many patients still develop GVHD, and glucocorticoids are an effective treatment for both aGVHD and cGVHD (83). But glucocorticoids can cause side effects such as osteoporosis, susceptibility to infection, increased blood sugar and blood pressure, necrosis of femoral head and so on. In summary, GVHD affects the quality of survival, mortality in allo-HSCT patients. Gut microbiota plays an important role in the development and treatment of GVHD (84).

Gut microbiota correlates with the development of GVHD. In 1974, van Bekkum DW et al. found that GVHD occurred in mice treated with lethal total body irradiation and allo-HSCT, with a mortality rate of 95% within 100 days, whereas mice treated similarly in a sterile state or given a colonization-resistant flora had almost no GVHD (85). This study confirmed in an animal model that the microbiome is associated with GVHD. In a study of mice, it was found that the gut microbiota diversity of mice with GVHD decreased significantly during the first 2 weeks of bone marrow transplantation, and in terms of changes in bacterial subgroups, the number of Lactobacillus increased significantly in the ileum, while the number of Clostridium and other Firmicutes decreased significantly (86). The authors also found that removal of Lactobacillus prior to bone marrow transplantation aggravated GVHD in mice and that reintroduction of a dominant species of Lactobacillus mediated protection against GVHD (86).

The presence of disruption of the gut microbiota early in bone marrow transplantation is also a potential risk factor for GVHD (86). We already know that SCFAs can reduce DNA damage in blood and gastrointestinal tissues during radiation damage (31), and SCFAs inhibit histone deacetylation (45). SCFAs mainly include formate, acetate, propionate and butyrate (87). Mathewson ND et al. analyzed SCFAs produced by the microbiome of allo-HSCT mice and found that only butyrate was significantly reduced in the intestinal tissues of transplanted recipients, resulting in reduced levels of histone deacetylation in small intestinal epithelial cells. In contrast, increasing the level of butyrate in the small intestine restored histone deacetylation, protected small intestinal epithelial cells, and reduced the severity of GVHD (88). The mechanism by which butyrate protects small intestinal epithelial cells may be through the regulation of histone deacetylation to increase anti-apoptotic genes. In butyrate-treated small intestinal epithelial cells, Bak1 and Bax were significantly reduced and the anti-apoptotic protein BCL-B was significantly increased (88). In the study of SCFAs and cGVHD, higher SCFAs (mainly butyrate and propionate) in the plasma of patients at 100 days post-transplantation were associated with decreased cGVHD (89). Golob JL et al. prospectively collected 669 stool samples from 66 allo-HSCT patients, once a week from pre-transplant to 100 days post-transplant. Their analysis revealed that the presence of Lachnospiraceae in stool was negatively associated with GVHD when neutrophils recovered after transplantation, whereas the presence of oral Actinobacteria and oral Firmicutes was positively associated with GVHD (90). A study by Han L et al. found more gut microbiota diversity in the non-aGVHD group than in the aGVHD group (91), they evaluated the gut microbiota to predict GVHD and found that Lachnospiraceae and Peptostreptococcaceae were negatively associated with GVHD, while Enterobacteriaceae were positively associated with aGVHD (91). Lachnospiraceae was negatively correlated with severe aGVHD, and levels of all major SFCAs were significantly decreased in patients with severe aGVHD, with a 75.8% decrease in acetate, a 95.8% decrease in propionate, and a 94.6% decrease in butyrate, while propionate levels were retained in patients with mild aGVHD. Butyrate was severely reduced in all gastrointestinal stages of aGVHD (92). Patients with ≤10% Lachnospiraceae in stool specimens at transplant pretreatment were significant contributors to overall mortality, and reduced gut microbiota diversity at day 10 post-transplant significantly increased the risk of GVHD (93). These studies all confirmed that abundant Lachnospiraceae and intact gut microbiota diversity were negatively correlated with GVHD (90–93). Lachnospiraceae-dominant gut microbiota mice also can withstand high doses of radiation (31), Lachnospiraceae and SCFA (propionate) are used to modulate host resistance to high doses of radiation by promoting hematopoiesis and gastrointestinal recovery (31).

Another product of the gut microbiota, indole, has also been shown to improve GVHD. Indole is produced by certain intestinal microflora (e.g., Escherichia coli, Lactobacillus, Bacteroides) (94). Indole-3-carbaldehyde (ICA) is an exogenous indole derivative. ICA upregulated genes associated with type I interferon (IFN1) responses, limited intestinal epithelial damage, reduced inflammatory cytokine production, and decreased GVHD pathology and GVHD mortality without affecting donor T cell-mediated graft-versus-leukemia responses (95). Although SCFAs and indoles, metabolites of the gut microbiota, have been shown to reduce the severity of GVHD, another circulating gut microbial metabolite, TMA N-oxide (TMAO), has been reported to enhance M1 macrophage polarization, and polarized inflammatory macrophages establish an environment for t helper type 1 (Th1) and Th17 responses, thereby increasing GVHD (96).

The use of broad-spectrum antibiotics can increase the severity of GVHD. In a mouse model of allo-HSCT, treatment with imipenem-cilastatin was found to disrupt intestinal microbiota and increase the severity of GVHD (mainly increased GVHD pathology in the colon) in mice with GVHD, resulting in increased mortality (97). However, if the mice did not develop GVHD, treatment with imipenem-cilastatin did not affect the survival of the mice (97). Why did the use of imipenem-cilastatin lead to increased severity of GVHD in mice? Further studies revealed that in these mice, donor effector CD4+ T cells (CD25+FoxP3-) and interleukin-23 (IL-23) were increased and granulocytes were infiltrated in their colon (97). AL patients treated with intensive chemotherapy and broad-spectrum antibiotics destroy the diversity of the intestinal microbiota (98). The use of autologous fecal microbiota transfer (AFMT) restores intestinal flora diversity and is safe for immunocompromised patients (98). Whether AFMT can improve GVHD requires further study.

Gut microbiota correlates with the prognosis of allo-HSCT. Kusakabe S et al. studied the gut microbiota of normal, autologous HSCT patients and allo-HSCT patients, and they found that in allo-HSCT patients with skewed microbiota had a higher frequency of more complications and death after transplantation than the subgroup with preserved microbiota composition (93). The diversity of the gut microbiota is an independent predictor of mortality in allo-HSCT patients, Taur Y collected stools from 80 allo-HSCT patients for analysis, and the percentage of patients maintaining high, intermediate, and low diversity of the gut microbiota was 26 (32.5%), 20 (25.0%), and 34 (42.5%), and their 3-year overall survival rates were 67%, 60% and 36%, respectively (99). Peled JU et al. analyzed 8767 stool samples from 1362 patients who received allo-HSCT from a total of four transplant centers in the United States, Germany, and Japan and found that higher gut microbiota diversity at the time of neutrophil engraftment was associated with lower patient mortality, patients with low gut microbiota diversity were positively associated with transplant-related mortality and a high risk of GVHD (100). A study on gut microbiota and pulmonary complications after allo-HSCT found that low gut microbiota diversity was associated with PCs occurring preengraftment, whereas γ-proteobacteria dominant gut flora was associated with PCs postengraftment (101). PCs postengraftment and γ-proteobacteria dominant gut flora were associated with mortality (101). In a study of a total of four bone marrow transplant centers from the United States, Japan, and Germany, the investigators defined Enterococcal dominance as having a relative genic abundance of ≥0.3 in any fecal sample (102). The incidence of Enterococcus dominance was high at all centers, with 441 of 1101 patients (40.1%) at Memorial Sloan Kettering Cancer Center (MSKCC) and 103 of 224 patients (46.0%) at the other 3 centers having E. faecium dominance between day -20 and day +80 of allo-HSCT (102). Early post-transplant (day 0 to + 21) Enterococcus dominance significantly reduced overall survival and increased GVHD-related mortality, increasing the risk of severe aGVHD (102). In a mouse model of allo-HSCT, an increase in Enterococcus in the feces of mice was also positively associated with GVHD, and the absence of lactose in the diet significantly reduced the colonization of Enterococcus after transplantation and attenuated GVHD (102). Lactose-free diets may be used in clinical settings to attenuate the growth of pathogens such as enterococci and improve clinical outcomes.

Interestingly, it has been shown that the relative abundance of Eubacterium hallii is higher in minimal residual disease (MRD)-negative multiple myeloma patients compared to MRD-positive patients (103). Whether the relative abundance of gut microbiota is associated with risk stratification of AL and MRD has not been studied, which warrants further research.

FMT is a treatment for multidrug-resistant bacteria colonization and GVHD in patients undergoing allo-HSCT. Battipaglia G et al. performed FMT in 10 patients colonized by multidrug resistance; eight of these patients were colonized by carbapenemase-producing bacteria and two by vancomycin-resistant enterococci; four of them underwent FMT before allo-HSCT and the other six underwent FMT after allo-HSCT; seven of the 10 patients achieved decolonization (104). We already know that disruption of gut microbiota diversity can exacerbate GVHD, but patients are in a state of severe immunosuppression after allo-HSCT, and performing FMT on them may be at potential risk for microbial infection. Kakihana K et al. performed FMT on one glucocorticoid-dependent and three glucocorticoid-resistant patients with gut aGVHD, and none of the patients experienced serious adverse effects (105). All four patients responded to FMT, with three complete responses and one partial response (105). DeFilipp Z et al. performed FMT on 13 patients at a median of 27 days (19-45) after allo-HSCT, and analysis of the patients’ fecal microbiota composition and urinary 3-indoxyl sulfate concentration showed that FMT reconstructed the diversity of the patients’ gut microbiota (106). We summarized recent studies on gut microbiota and GVHD (**Table 1**).

**Table 1:** Mechanisms by which the gut microbiota affects GVHD.

Influence of gut microbiota on GVHD	Mechanisms	References
Lactobacillus shows improvement in GVHD	–	(86)
Disruption of gut microbiota diversity aggravates GVHD	–	(86, 91, 93)
Butyrate, a metabolite of the gut microbiota, attenuates GVHD	Butyrate improved IEC junctional integrity, decreased apoptosis	(88, 89)
Lachnospiraceae is negatively associated with GVHD	–	(90–93)
Indole, a metabolite of the gut microbiota, improves GVHD	Indole upregulates genes associated with IFN1 response, limits intestinal epithelial damage, reduces inflammatory cytokine production, and decreases GVHD pathology and GVHD mortality.	(95)
GVHD progression was induced by TMAO, a circulating gut microbial metabolite	TMAO enhances M1 macrophage polarization and establishes an environment for Th1 and Th17 responses	(96)
Antibiotic use alters the composition of the gut microbiota and increases GVHD	Loss of protective colonic mucosa and impairment of intestinal barrier function due to antibiotics	(97)
Obesity aggravates GVHD by affecting the gut microbiota	Obesity reduces the diversity of the gut microbiota and reduces Clostridiaceae abundance, leading to increased intestinal permeability, transintestinal transit of endotoxins, and radiation-induced gastrointestinal damage in mice	(107)
The microbiota controls MHC-II at the pre-transplant IEC	Microbiota depletion inhibits IL-12/23p40 production by ileal macrophages, IL-12/23p40 prevents upregulation of MHC class II cells on IECs and initiation of lethal GVHD in the gastrointestinal tract	(108)
The gut microbiota metabolite sensor G-protein-coupled receptor 43 (GPR43) attenuates gastrointestinal GVHD	GVHD protection by SCFAs requires GPR43-mediated ERK phosphorylation and activation of NLRP3 inflammasome in host non-hematopoietic target tissues	(109)

A study analyzing the gut microbiota of 541 patients undergoing allo-HSCT found that the cumulative incidence of recurrence/progression at 2 years was 19.8% and 33.8% in patients with and without Eubacterium limosum, respectively, with higher abundance of Eubacterium limosum being associated with fewer recurrences (110). Eubacterium limosum may serve as a biomarker for allo-HSCT to predict recurrence, and the exact mechanism by which it reduces recurrence needs to be confirmed by further studies.

In general, the gut microbiota may play an important role in the pathogenesis, progression and treatment of AL by influencing human immunity. The gut microbiota has been found to resist damage from radiation and benzene, which may cause AL. The gut microbiota is also closely related to the body’s normal immunity, and in AL, the diversity of the gut microbiota is associated with multiple infections. When AL patients undergo chemotherapy, the gut microbiota is affected and, in turn, the gut microbiota affects the efficacy of multiple chemotherapeutic agents. The diversity of the gut microbiota and its metabolites are strongly associated with the occurrence of GVHD, mortality and relapse of allo-HSCT. More studies can be conducted in the future to focus on the relationship between the gut microbiota and AL, especially that the gut microbiota improves the treatment outcome and prognosis of AL patients by regulating human immunity.

## Author Contributions

TM and YC wrote the manuscript. L-JL and L-SZ wrote and critically revised the manuscript. All authors contributed to the article and approved the submitted version.

## Funding

The present study was supported by grants from the National Natural Science Foundation of China (Grant No. 31660112); The Science and Technique Innovation Project of Lanzhou University Second Hospital (CY2017-ZD04 and CY2019-MS14); The Research Foundation of The Affiliated Hospital of Southwest Medical University (Grant No. 15015); The Research Foundation of Southwest Medical University (2017-ZRQN-092); and The Talent Innovation and Entrepreneurship Project of Lanzhou Science and Technology Bureau (2020-RC-48).

## Conflict of Interest

The authors declare that the research was conducted in the absence of any commercial or financial relationships that could be construed as a potential conflict of interest.

## References

[1] Wen Y Jin R Chen H. Interactions Between Gut Microbiota and Acute Childhood Leukemia. Front Microbiol (2019) 10:1300. 10.3389/fmicb.2019.01300 31275258 PMC6593047

[2] Bray F Ferlay J Soerjomataram I Siegel RL Torre LA Jemal A. Global Cancer Statistics 2018: GLOBOCAN Estimates of Incidence and Mortality Worldwide for 36 Cancers in 185 Countries. CA Cancer J Clin (2018) 68(6):394–424. 10.3322/caac.21492 [Published Correction Appears in CA Cancer J Clin. 2020 Jul;70(4):313]. 30207593

[3] Steliarova-Foucher E Colombet M Ries LAG Moreno F Dolya A Bray F. International Incidence of Childhood Cancer, 2001-10: A Population-Based Registry Study. Lancet Oncol (2017) 18(6):719–31. 10.1016/S1470-2045(17)30186-9 [Published Correction Appears in Lancet Oncol. 2017 Jun;18(6):e301]. PMC5461370 28410997

[4] Jabbour E O’Brien S Konopleva M Kantarjian H. New Insights Into the Pathophysiology and Therapy of Adult Acute Lymphoblastic Leukemia. Cancer (2015) 121(15):2517–28. 10.1002/cncr.29383 PMC11726371 25891003

[5] Yang X Wang J. Precision Therapy for Acute Myeloid Leukemia. J Hematol Oncol (2018) 11(1):3. 10.1186/s13045-017-0543-7 29301553 PMC5755341

[6] McNerney ME Godley LA Le Beau MM. Therapy-Related Myeloid Neoplasms: When Genetics and Environment Collide. Nat Rev Cancer (2017) 17(9):513–27. 10.1038/nrc.2017.60 PMC5946699 28835720

[7] Short NJ Rytting ME Cortes JE. Acute Myeloid Leukaemia. Lancet (2018) 392(10147):593–606. 10.1016/S0140-6736(18)31041-9 30078459 PMC10230947

[8] Williams P Basu S Garcia-Manero G Hourigan CS Oetjen KA Cortes JE. The Distribution of T-cell Subsets and the Expression of Immune Checkpoint Receptors and Ligands in Patients With Newly Diagnosed and Relapsed Acute Myeloid Leukemia. Cancer (2019) 125(9):1470–81. 10.1002/cncr.31896 PMC6467779 30500073

[9] Le Dieu R Taussig DC Ramsay AG Mitter R Miraki-Moud F Fatah R. Peripheral Blood T Cells in Acute Myeloid Leukemia (AML) Patients at Diagnosis Have Abnormal Phenotype and Genotype and Form Defective Immune Synapses With AML Blasts. Blood (2009) 114(18):3909–16. 10.1182/blood-2009-02-206946 PMC2773481 19710498

[10] Ustun C Miller JS Munn DH Weisdorf DJ Blazar BR. Regulatory T Cells in Acute Myelogenous Leukemia: Is it Time for Immunomodulation? Blood (2011) 118(19):5084–95. 10.1182/blood-2011-07-365817 PMC3217399 21881045

[11] Szczepanski MJ Szajnik M Czystowska M Mandapathil M Strauss L Welsh A. Increased Frequency and Suppression by Regulatory T Cells in Patients With Acute Myelogenous Leukemia. Clin Cancer Res (2009) 15(10):3325–32. 10.1158/1078-0432.CCR-08-3010 PMC3700356 19417016

[12] Xu X Sun Q Liang X Chen Z Zhang X Zhou X. Mechanisms of Relapse After CD19 Car T-Cell Therapy for Acute Lymphoblastic Leukemia and Its Prevention and Treatment Strategies. Front Immunol (2019) 10:2664. 10.3389/fimmu.2019.02664 31798590 PMC6863137

[13] Adak A Khan MR. An Insight Into Gut Microbiota and Its Functionalities. Cell Mol Life Sci (2019) 76(3):473–93. 10.1007/s00018-018-2943-4 PMC11105460 30317530

[14] Milani C Duranti S Bottacini F Casey E Turroni F Mahony J. The First Microbial Colonizers of the Human Gut: Composition, Activities, and Health Implications of the Infant Gut Microbiota. Microbiol Mol Biol Rev (2017) 81(4):e00036–17. 10.1128/MMBR.00036-17 PMC5706746 29118049

[15] Sánchez B Delgado S Blanco-Míguez A Lourenço A Gueimonde M Margolles A. Probiotics, Gut Microbiota, and Their Influence on Host Health and Disease. Mol Nutr Food Res (2017) 61(1):10.1002/mnfr.201600240. 10.1002/mnfr.201600240 27500859

[16] Pickard JM Zeng MY Caruso R Núñez G. Gut Microbiota: Role in Pathogen Colonization, Immune Responses, and Inflammatory Disease. Immunol Rev (2017) 279(1):70–89. 10.1111/imr.12567 28856738 PMC5657496

[17] Sethi V Kurtom S Tarique M Lavania S Malchiodi Z Hellmund L. Gut Microbiota Promotes Tumor Growth in Mice by Modulating Immune Response. Gastroenterology (2018) 155(1):33–37.e6. 10.1053/j.gastro.2018.04.001 29630898 PMC6035070

[18] Jiang C Li G Huang P Liu Z Zhao B. The Gut Microbiota and Alzheimer’s Disease. J Alzheimers Dis (2017) 58(1):1–15. 10.3233/JAD-161141 28372330

[19] Velmurugan G Ramprasath T Gilles M Swaminathan K Ramasamy S. Gut Microbiota, Endocrine-Disrupting Chemicals, and the Diabetes Epidemic. Trends Endocrinol Metab (2017) 28(8):612–25. 10.1016/j.tem.2017.05.001 28571659

[20] Greaves M. A Causal Mechanism for Childhood Acute Lymphoblastic Leukaemia [Published Correction Appears in Nat Rev Cancer. Nat Rev Cancer (2018) 18(8):471–84. 10.1038/s41568-018-0015-6 PMC6986894 29784935

[21] Hein D Dreisig K Metzler M Izraeli S Schmiegelow K Borkhardt A. The Preleukemic TCF3-PBX1 Gene Fusion can be Generated In Utero and Is Present in ≈0.6% of healthy newborns. Blood (2019) 134(16):1355–8. 10.1182/blood.2019002215 PMC7005361 31434706

[22] Schäfer D Olsen M Lähnemann D. Five Percent of Healthy Newborns Have an ETV6-RUNX1 Fusion as Revealed by DNA-Based GIPFEL Screening. Blood (2018) 131(7):821–6. 10.1182/blood-2017-09-808402 PMC5909885 29311095

[23] Rodríguez-Hernández G Hauer J Martín-Lorenzo A Schäfer D Bartenhagen C García-Ramírez I. Infection Exposure Promotes ETV6-RUNX1 Precursor B-Cell Leukemia Via Impaired H3k4 Demethylases. Cancer Res (2017) 77(16):4365–77. 10.1158/0008-5472.CAN-17-0701 28630052

[24] Martín-Lorenzo A Hauer J Vicente-Dueñas C Auer F González-Herrero I García-Ramírez I. Infection Exposure Is a Causal Factor in B-Cell Precursor Acute Lymphoblastic Leukemia as a Result of Pax5-Inherited Susceptibility. Cancer Discov (2015) 5(12):1328–43. 10.1158/2159-8290.CD-15-0892 26408659

[25] Vicente-Dueñas C Janssen S Oldenburg M Auer F González-Herrero I Casado-García A. An Intact Gut Microbiome Protects Genetically Predisposed Mice Against Leukemia. Blood (2020) 136(18):2003–17. 10.1182/blood.2019004381 PMC7694022 32911536

[26] Liu X Zou Y Ruan M Chang L Chen X Wang S. Pediatric Acute Lymphoblastic Leukemia Patients Exhibit Distinctive Alterations in the Gut Microbiota. Front Cell Infect Microbiol (2020) 10:558799. 10.3389/fcimb.2020.558799 33178621 PMC7596659

[27] Song Y Gyarmati P. Microbiota Changes in a Pediatric Acute Lymphocytic Leukemia Mouse Model. Microbiologyopen (2020) 9(3):e982. 10.1002/mbo3.982 31884727 PMC7066458

[28] Strick R Strissel PL Borgers S Smith SL Rowley JD. Dietary Bioflavonoids Induce Cleavage in the MLL Gene and may Contribute to Infant Leukemia. Proc Natl Acad Sci USA (2000) 97(9):4790–5. 10.1073/pnas.070061297 PMC18311 10758153

[29] Østgård LSG Nørgaard M Pedersen L Østgård RD Medeiros BC Overgaard UM. Autoimmune Diseases, Infections, Use of Antibiotics and the Risk of Acute Myeloid Leukaemia: A National Population-Based Case-Control Study. Br J Haematol (2018) 181(2):205–14. 10.1111/bjh.15163 29504124

[30] Little MP Wakeford R Borrego D French B Zablotska LB Adams MJ. Leukaemia and Myeloid Malignancy Among People Exposed to Low Doses (<100 mSv) of Ionising Radiation During Childhood: A Pooled Analysis of Nine Historical Cohort Studies. Lancet Haematol (2018) 5(8):e346–58. 10.1016/S2352-3026(18)30092-9 PMC6130888 30026010

[31] Guo H Chou WC Lai Y Liang K Tam JW Brickey WJ. Multi-Omics Analyses of Radiation Survivors Identify Radioprotective Microbes and Metabolites. Science (2020) 370(6516):eaay9097. 10.1126/science.aay9097 33122357 PMC7898465

[32] Ciorba MA Riehl TE Rao MS Moon C Ee X Nava GM. Lactobacillus Probiotic Protects Intestinal Epithelium From Radiation Injury in a TLR-2/cyclo-oxygenase-2-dependent Manner. Gut (2012) 61(6):829–38. 10.1136/gutjnl-2011-300367 PMC3345937 22027478

[33] Cui M Xiao H Li Y Zhou L Zhao S Luo D. Faecal Microbiota Transplantation Protects Against Radiation-Induced Toxicity. EMBO Mol Med (2017) 9(4):448–61. 10.15252/emmm.201606932 PMC5376756 28242755

[34] Loomis D Guyton KZ Grosse Y El Ghissassi F Bouvard V Benbrahim-Tallaa L. Carcinogenicity of Benzene. Lancet Oncol (2017) 18(12):1574–5. 10.1016/S1470-2045(17)30832-X 29107678

[35] Houot J Marquant F Goujon S Faure L Honoré C Roth MH. Residential Proximity to Heavy-Traffic Roads, Benzene Exposure, and Childhood Leukemia-The GEOCAP Study, 2002-2007. Am J Epidemiol (2015) 182(8):685–93. 10.1093/aje/kwv111 26377958

[36] Sun R Xu K Ji S Pu Y Man Z Ji J. Benzene Exposure Induces Gut Microbiota Dysbiosis and Metabolic Disorder in Mice. Sci Total Environ (2020) 705:135879. 10.1016/j.scitotenv.2019.135879 31972927

[37] Macpherson AJ Harris NL. Interactions Between Commensal Intestinal Bacteria and the Immune System. Nat Rev Immunol (2004) 4(6):478–85. 10.1038/nri1373 15173836

[38] Tilg H Adolph TE Gerner RR Moschen AR. The Intestinal Microbiota in Colorectal Cancer. Cancer Cell (2018) 33(6):954–64. 10.1016/j.ccell.2018.03.004 29657127

[39] Li Q Jin M Liu Y Jin L. Gut Microbiota: Its Potential Roles in Pancreatic Cancer. Front Cell Infect Microbiol (2020) 10:572492. 10.3389/fcimb.2020.572492 33117731 PMC7575684

[40] Inaba H Pei D Wolf J Howard SC Hayden RT Go M. Infection-Related Complications During Treatment for Childhood Acute Lymphoblastic Leukemia. Ann Oncol (2017) 28(2):386–92. 10.1093/annonc/mdw557 PMC5834143 28426102

[41] Bochennek K Hassler A Perner C Gilfert J Schöning S Klingebiel T. Infectious Complications in Children With Acute Myeloid Leukemia: Decreased Mortality in Multicenter Trial AML-BFM 2004. Blood Cancer J (2016) 6(1):e382. 10.1038/bcj.2015.110 26771808 PMC4742627

[42] Chung H Pamp SJ Hill JA Surana NK Edelman SM Troy EB. Gut Immune Maturation Depends on Colonization With a Host-Specific Microbiota. Cell (2012) 149(7):1578–93. 10.1016/j.cell.2012.04.037 PMC3442780 22726443

[43] Pultz NJ Stiefel U Subramanyan S Helfand MS Donskey CJ. Mechanisms by Which Anaerobic Microbiota Inhibit the Establishment in Mice of Intestinal Colonization by Vancomycin-Resistant Enterococcus. J Infect Dis (2005) 191(6):949–56. 10.1086/428090 15717271

[44] Ford CD Coombs J Stofer MG Lopansri BK Webb BJ Ostronoff F. Decrease in Vancomycin-Resistant Enterococcus Colonization Associated With a Reduction in Carbapenem Use as Empiric Therapy for Febrile Neutropenia in Patients With Acute Leukemia. Infect Control Hosp Epidemiol (2019) 40(7):774–9. 10.1017/ice.2019.93 31046849

[45] Zhang S Dogan B Guo C Herlekar D Stewart K Scherl EJ. Short Chain Fatty Acids Modulate the Growth and Virulence of Pathosymbiont Escherichia Coli and Host Response. Antibiotics (Basel) (2020) 9(8):462. 10.3390/antibiotics9080462 PMC7460008 32751519

[46] Fachi JL Felipe JS Pral LP da Silva BK Corrêa RO de Andrade MCP. Butyrate Protects Mice From Clostridium Difficile-Induced Colitis Through an HIF-1-Dependent Mechanism. Cell Rep (2019) 27(3):750–761.e7. 10.1016/j.celrep.2019.03.054 30995474

[47] Josefsdottir KS Baldridge MT Kadmon CS King KY. Antibiotics Impair Murine Hematopoiesis by Depleting the Intestinal Microbiota. Blood (2017) 129(6):729–39. 10.1182/blood-2016-03-708594 PMC5301822 27879260

[48] Dodd D Spitzer MH Van Treuren W Merrill BD Hryckowian AJ Higginbottom SK. A Gut Bacterial Pathway Metabolizes Aromatic Amino Acids Into Nine Circulating Metabolites. Nature (2017) 551(7682):648–52. 10.1038/nature24661 PMC5850949 29168502

[49] Rashidi A Weisdorf DJ. Microbiota-Based Approaches to Mitigate Infectious Complications of Intensive Chemotherapy in Patients With Acute Leukemia. Transl Res (2020) 220:167–81. 10.1016/j.trsl.2020.03.011 PMC7605891 32275896

[50] Galloway-Peña JR Shi Y Peterson CB Sahasrabhojane P Gopalakrishnan V Brumlow CE. Gut Microbiome Signatures Are Predictive of Infectious Risk Following Induction Therapy for Acute Myeloid Leukemia. Clin Infect Dis (2020) 71(1):63–71. 10.1093/cid/ciz777 31436833 PMC7312220

[51] Galloway-Peña JR Smith DP Sahasrabhojane P Ajami NJ Wadsworth WD Daver NG. The Role of the Gastrointestinal Microbiome in Infectious Complications During Induction Chemotherapy for Acute Myeloid Leukemia. Cancer (2016) 122(14):2186–96. 10.1002/cncr.30039 PMC5574182 27142181

[52] Hakim H Dallas R Wolf J Tang L Schultz-Cherry S Darling V. Gut Microbiome Composition Predicts Infection Risk During Chemotherapy in Children With Acute Lymphoblastic Leukemia. Clin Infect Dis (2018) 67(4):541–8. 10.1093/cid/ciy153 PMC6070042 29518185

[53] Nearing JT Connors J Whitehouse S Van Limbergen J Macdonald T Kulkarni K. Infectious Complications Are Associated With Alterations in the Gut Microbiome in Pediatric Patients With Acute Lymphoblastic Leukemia. Front Cell Infect Microbiol (2019) 9:28. 10.3389/fcimb.2019.00028 30838178 PMC6389711

[54] Ralls MW Demehri FR Feng Y Woods Ignatoski KM Teitelbaum DH. Enteral Nutrient Deprivation in Patients Leads to a Loss of Intestinal Epithelial Barrier Function. Surgery (2015) 157(4):732–42. 10.1016/j.surg.2014.12.004 PMC4369405 25704423

[55] Reyna-Figueroa J Barrón-Calvillo E García-Parra C Galindo-Delgado P Contreras-Ochoa C Lagunas-Martínez A. Probiotic Supplementation Decreases Chemotherapy-Induced Gastrointestinal Side Effects in Patients With Acute Leukemia. J Pediatr Hematol Oncol (2019) 41(6):468–72. 10.1097/MPH.0000000000001497 31033786

[56] Suez J Zmora N Zilberman-Schapira G Mor U Dori-Bachash M Bashiardes S. Post-Antibiotic Gut Mucosal Microbiome Reconstitution Is Impaired by Probiotics and Improved by Autologous Fmt. Cell (2018) 174(6):1406–23.e16. 10.1016/j.cell.2018.08.047 30193113

[57] Koyama S Fujita H Shimosato T Kamijo A Ishiyama Y Yamamoto E. Septicemia From Lactobacillus Rhamnosus GG, From a Probiotic Enriched Yogurt, in a Patient With Autologous Stem Cell Transplantation. Probiotics Antimicrob Proteins (2019) 11(1):295–8. 10.1007/s12602-018-9399-6 29455334

[58] Avcin SL Pokorn M Kitanovski L Premru MM Jazbec J. Bifidobacterium Breve Sepsis in Child With High-Risk Acute Lymphoblastic Leukemia. Emerg Infect Dis (2015) 21(9):1674–5. 10.3201/eid2109.150097 PMC4550169 26291071

[59] Rashidi A Kaiser T Graiziger C Holtan SG Rehman TU Weisdorf DJ. Specific Gut Microbiota Changes Heralding Bloodstream Infection and Neutropenic Fever During Intensive Chemotherapy. Leukemia (2020) 34(1):312–6. 10.1038/s41375-019-0547-0 31439944

[60] Alexander JL Wilson ID Teare J Marchesi JR Nicholson JK Kinross JM. Gut Microbiota Modulation of Chemotherapy Efficacy and Toxicity. Nat Rev Gastroenterol Hepatol (2017) 14(6):356–65. 10.1038/nrgastro.2017.20 28270698

[61] Viaud S Saccheri F Mignot G Yamazaki T Daillère R Hannani D. The Intestinal Microbiota Modulates the Anticancer Immune Effects of Cyclophosphamide. Science (2013) 342(6161):971–6. 10.1126/science.1240537 PMC4048947 24264990

[62] Liu T Wu Y Wang L Pang X Zhao L Yuan H. A More Robust Gut Microbiota in Calorie-Restricted Mice Is Associated With Attenuated Intestinal Injury Caused by the Chemotherapy Drug Cyclophosphamide. mBio (2019) 10(2):e02903–18. 10.1128/mBio.02903-18 PMC6414708 30862756

[63] Sakura T Hayakawa F Sugiura I Murayama T Imai K Usui N. High-Dose Methotrexate Therapy Significantly Improved Survival of Adult Acute Lymphoblastic Leukemia: A Phase III Study by JALSG. Leukemia (2018) 32(3):626–32. 10.1038/leu.2017.283。 28914260

[64] Larson RA. Managing CNS Disease in Adults With Acute Lymphoblastic Leukemia. Leuk Lymphoma (2018) 59(1):3–13. 10.1080/10428194.2017.1326597 28535095

[65] Friedman B Cronstein B. Methotrexate Mechanism in Treatment of Rheumatoid Arthritis. Joint Bone Spine (2019) 86(3):301–7. 10.1016/j.jbspin.2018.07.004 PMC6360124 30081197

[66] Kolli VK Abraham P Isaac B Kasthuri N. Preclinical Efficacy of Melatonin to Reduce Methotrexate-Induced Oxidative Stress and Small Intestinal Damage in Rats. Dig Dis Sci (2013) 58(4):959–69. 10.1007/s10620-012-2437-4 23053903

[67] Ben-Lulu S Pollak Y Mogilner J Bejar J G Coran A Sukhotnik I. Dietary Transforming Growth Factor-Beta 2 (TGF-β2) Supplementation Reduces Methotrexate-Induced Intestinal Mucosal Injury in a Rat. PloS One (2012) 7(9):e45221. 10.1371/journal.pone.0045221 22984629 PMC3440324

[68] Zhou B Xia X Wang P Chen S Yu C Huang R. Induction and Amelioration of Methotrexate-Induced Gastrointestinal Toxicity Are Related to Immune Response and Gut Microbiota. EBioMedicine (2018) 33:122–33. 10.1016/j.ebiom.2018.06.029 PMC6085585 30049384

[69] Letertre MPM Munjoma N Wolfer K Pechlivanis A McDonald JAK Hardwick RN. A Two-Way Interaction Between Methotrexate and the Gut Microbiota of Male Sprague-Dawley Rats. J Proteome Res (2020) 19(8):3326–39. 10.1021/acs.jproteome.0c00230 PMC7426014 32544340

[70] Nayak RR Alexander M Deshpande I Stapleton-Gray K Rimal B Patterson AD. Methotrexate Impacts Conserved Pathways in Diverse Human Gut Bacteria Leading to Decreased Host Immune Activation. Cell Host Microbe (2021) 29(3):362–77.e11. 10.1016/j.chom.2020.12.008 PMC7954989 33440172

[71] Farkona S Diamandis EP Blasutig IM. Cancer Immunotherapy: The Beginning of the End of Cancer? BMC Med (2016) 14:73. 10.1186/s12916-016-0623-5 27151159 PMC4858828

[72] Zhou Q Munger ME Highfill SL Tolar J Weigel BJ Riddle M. Program Death-1 Signaling and Regulatory T Cells Collaborate to Resist the Function of Adoptively Transferred Cytotoxic T Lymphocytes in Advanced Acute Myeloid Leukemia. Blood (2010) 116(14):2484–93. 10.1182/blood-2010-03-275446 PMC2953885 20570856

[73] Daver N Garcia-Manero G Basu S Boddu PC Alfayez M Cortes JE. Efficacy, Safety, and Biomarkers of Response to Azacitidine and Nivolumab in Relapsed/Refractory Acute Myeloid Leukemia: A Nonrandomized, Open-Label, Phase II Study. Cancer Discovery (2019) 9(3):370–83. 10.1158/2159-8290.CD-18-0774 PMC6397669 30409776

[74] Routy B Le Chatelier E Derosa L Duong CPM Alou MT Daillère R. Gut Microbiome Influences Efficacy of PD-1-based Immunotherapy Against Epithelial Tumors. Science (2018) 359(6371):91–7. 10.1126/science.aan3706 29097494

[75] Matson V Fessler J Bao R Chongsuwat T Zha Y Alegre ML. The Commensal Microbiome Is Associated With Anti-PD-1 Efficacy in Metastatic Melanoma Patients. Science (2018) 359(6371):104–8. 10.1126/science.aao3290 PMC6707353 29302014

[76] Gopalakrishnan V Spencer CN Nezi L Reuben A Andrews MC Karpinets TV. Gut Microbiome Modulates Response to Anti-PD-1 Immunotherapy in Melanoma Patients. Science (2018) 359(6371):97–103. 10.1126/science.aan4236 29097493 PMC5827966

[77] Zheng Y Wang T Tu X Huang Y Zhang H Tan D. Gut Microbiome Affects the Response to Anti-PD-1 Immunotherapy in Patients With Hepatocellular Carcinoma. J Immunother Cancer (2019) 7(1):193. 10.1186/s40425-019-0650-9 31337439 PMC6651993

[78] Lee CJ Savani BN Mohty M Gorin NC Labopin M Ruggeri A. Post-Remission Strategies for the Prevention of Relapse Following Allogeneic Hematopoietic Cell Transplantation for High-Risk Acute Myeloid Leukemia: Expert Review From the Acute Leukemia Working Party of the European Society for Blood and Marrow Transplantation. Bone Marrow Transplant (2019) 54(4):519–30. 10.1038/s41409-018-0286-2 30104717

[79] Fredricks DN. The Gut Microbiota and Graft-Versus-Host Disease. J Clin Invest (2019) 129(5):1808–17. 10.1172/JCI125797 PMC6486325 31042160

[80] Ferrara JL Levine JE Reddy P Holler E. Graft-Versus-Host Disease. Lancet (2009) 373(9674):1550–61. 10.1016/S0140-6736(09)60237-3 PMC2735047 19282026

[81] Sarantopoulos S Cardones AR Sullivan KM. How I Treat Refractory Chronic Graft-Versus-Host Disease. Blood (2019) 133(11):1191–200. 10.1182/blood-2018-04-785899 PMC6418480 30674472

[82] Zeiser R Blazar BR. Acute Graft-versus-Host Disease - Biologic Process, Prevention, and Therapy. N Engl J Med (2017) 377(22):2167–79. 10.1056/NEJMra1609337 PMC6034180 29171820

[83] Jacobsohn DA Vogelsang GB. Acute Graft Versus Host Disease. Orphanet J Rare Dis (2007) 2:35. 10.1186/1750-1172-2-35 17784964 PMC2018687

[84] Staffas A Burgos da Silva M van den Brink MR. The Intestinal Microbiota in Allogeneic Hematopoietic Cell Transplant and Graft-Versus-Host Disease. Blood (2017) 129(8):927–33. 10.1182/blood-2016-09-691394 [published correction appears in Blood. 2017 Apr 13;129(15):2204]. PMC5324712 27940475

[85] van Bekkum DW Roodenburg J Heidt PJ van der Waaij D. Mitigation of Secondary Disease of Allogeneic Mouse Radiation Chimeras by Modification of the Intestinal Microflora. J Natl Cancer Inst (1974) 52(2):401–4. 10.1093/jnci/52.2.401 4150164

[86] Jenq RR Ubeda C Taur Y Menezes CC Khanin R Dudakov JA. Regulation of Intestinal Inflammation by Microbiota Following Allogeneic Bone Marrow Transplantation. J Exp Med (2012) 209(5):903–11. 10.1084/jem.20112408 PMC3348096 22547653

[87] Morrison DJ Preston T. Formation of Short Chain Fatty Acids by the Gut Microbiota and Their Impact on Human Metabolism. Gut Microbes (2016) 7(3):189–200. 10.1080/19490976.2015.1134082 26963409 PMC4939913

[88] Mathewson ND Jenq R Mathew AV Koenigsknecht M Hanash A Toubai T. Gut Microbiome-Derived Metabolites Modulate Intestinal Epithelial Cell Damage and Mitigate Graft-Versus-Host Disease. Nat Immunol (2016) 17(5):505–13. 10.1038/ni.3400 [published correction appears in Nat Immunol. 2016 Sep 20;17 (10 ):1235]. PMC4836986 26998764

[89] Markey KA Schluter J Gomes ALC Littmann ER Pickard AJ Taylor BP. The Microbe-Derived Short-Chain Fatty Acids Butyrate and Propionate Are Associated With Protection From Chronic GVHD. Blood (2020) 136(1):130–6. 10.1182/blood.2019003369 PMC7332893 32430495

[90] Golob JL Pergam SA Srinivasan S Fiedler TL Liu C Garcia K. Stool Microbiota at Neutrophil Recovery Is Predictive for Severe Acute Graft vs Host Disease After Hematopoietic Cell Transplantation. Clin Infect Dis (2017) 65(12):1984–91. 10.1093/cid/cix699 PMC5850019 29020185

[91] Han L Zhao K Li Y Han H Zhou L Ma P. A Gut Microbiota Score Predicting Acute Graft-Versus-Host Disease Following Myeloablative Allogeneic Hematopoietic Stem Cell Transplantation. Am J Transplant (2020) 20(4):1014–27. 10.1111/ajt.15654 PMC7154648 31605563

[92] Payen M Nicolis I Robin M Michonneau D Delannoye J Mayeur C. Functional and Phylogenetic Alterations in Gut Microbiome Are Linked to Graft-Versus-Host Disease Severity. Blood Adv (2020) 4(9):1824–32. 10.1182/bloodadvances.2020001531 PMC7218439 32353108

[93] Kusakabe S Fukushima K Maeda T Motooka D Nakamura S Fujita J. Pre- and Post-Serial Metagenomic Analysis of Gut Microbiota as a Prognostic Factor in Patients Undergoing Haematopoietic Stem Cell Transplantation. Br J Haematol (2020) 188(3):438–49. 10.1111/bjh.16205 31566729

[94] Lee JH Wood TK Lee J. Roles of Indole as an Interspecies and Interkingdom Signaling Molecule. Trends Microbiol (2015) 23(11):707–18. 10.1016/j.tim.2015.08.001 26439294

[95] Swimm A Giver CR DeFilipp Z Rangaraju S Sharma A Ulezko Antonova A. Indoles Derived From Intestinal Microbiota Act Via Type I Interferon Signaling to Limit Graft-Versus-Host Disease. Blood (2018) 132(23):2506–19. 10.1182/blood-2018-03-838193 PMC6284212 30257880

[96] Wu K Yuan Y Yu H Dai X Wang S Sun Z. The Gut Microbial Metabolite Trimethylamine N-Oxide Aggravates GVHD by Inducing M1 Macrophage Polarization in Mice. Blood (2020) 136(4):501–15. 10.1182/blood.2019003990 PMC7378459 32291445

[97] Shono Y Docampo MD Peled JU Perobelli SM Velardi E Tsai JJ. Increased GVHD-related Mortality With Broad-Spectrum Antibiotic Use After Allogeneic Hematopoietic Stem Cell Transplantation in Human Patients and Mice. Sci Transl Med (2016) 8(339):339ra71. 10.1126/scitranslmed.aaf2311 PMC4991773 27194729

[98] Malard F Vekhoff A Lapusan S Isnard F D'incan-Corda E Rey J. Gut Microbiota Diversity After Autologous Fecal Microbiota Transfer in Acute Myeloid Leukemia Patients. Nat Commun (2021) 12(1):3084. 10.1038/s41467-021-23376-6 34035290 PMC8149453

[99] Taur Y Jenq RR Perales MA Littmann ER Morjaria S Ling L. The Effects of Intestinal Tract Bacterial Diversity on Mortality Following Allogeneic Hematopoietic Stem Cell Transplantation. Blood (2014) 124(7):1174–82. 10.1182/blood-2014-02-554725 PMC4133489 24939656

[100] Peled JU Gomes ALC Devlin SM Littmann ER Taur Y Sung AD. Microbiota as Predictor of Mortality in Allogeneic Hematopoietic-Cell Transplantation. N Engl J Med (2020) 382(9):822–34. 10.1056/NEJMoa1900623 PMC7534690 32101664

[101] Harris B Morjaria SM Littmann ER Geyer AI Stover DE Barker JN. Gut Microbiota Predict Pulmonary Infiltrates After Allogeneic Hematopoietic Cell Transplantation. Am J Respir Crit Care Med (2016) 194(4):450–63. 10.1164/rccm.201507-1491OC PMC5003327 26886180

[102] Stein-Thoeringer CK Nichols KB Lazrak A Docampo MD Slingerland AE Slingerland JB. Lactose Drives Enterococcus Expansion to Promote Graft-Versus-Host Disease. Science (2019) 366(6469):1143–9. 10.1126/science.aax3760 PMC7003985 31780560

[103] Pianko MJ Devlin SM Littmann ER Chansakul A Mastey D Salcedo M. Minimal Residual Disease Negativity in Multiple Myeloma Is Associated With Intestinal Microbiota Composition. Blood Adv (2019) 3(13):2040–4. 10.1182/bloodadvances.2019032276 PMC6616258 31289031

[104] Battipaglia G Malard F Rubio MT Ruggeri A Mamez AC Brissot E. Fecal Microbiota Transplantation Before or After Allogeneic Hematopoietic Transplantation in Patients With Hematologic Malignancies Carrying Multidrug-Resistance Bacteria. Haematologica (2019) 104(8):1682–8. 10.3324/haematol.2018.198549 PMC6669143 30733264

[105] Kakihana K Fujioka Y Suda W Najima Y Kuwata G Sasajima S. Fecal Microbiota Transplantation for Patients With Steroid-Resistant Acute Graft-Versus-Host Disease of the Gut. Blood (2016) 128(16):2083–8. 10.1182/blood-2016-05-717652 PMC5085256 27461930

[106] DeFilipp Z Peled JU Li S Mahabamunuge J Dagher Z Slingerland AE. Third-Party Fecal Microbiota Transplantation Following allo-HCT Reconstitutes Microbiome Diversity. Blood Adv (2018) 2(7):745–53. 10.1182/bloodadvances.2018017731 PMC5894265 29592876

[107] Khuat LT Le CT Pai CS Shields-Cutler RR Holtan SG Rashidi A. Obesity Induces Gut Microbiota Alterations and Augments Acute Graft-Versus-Host Disease After Allogeneic Stem Cell Transplantation. Sci Transl Med (2020) 12(571):eaay7713. 10.1126/scitranslmed.aay7713 33239390 PMC8525601

[108] Koyama M Mukhopadhyay P Schuster IS Henden AS Hülsdünker J Varelias A. Mhc Class Ii Antigen Presentation by the Intestinal Epithelium Initiates Graft-Versus-Host Disease and Is Influenced by the Microbiota. Immunity (2019) 51(5):885–898.e7. 10.1016/j.immuni.2019.08.011 31542340 PMC6959419

[109] Fujiwara H Docampo MD Riwes M Peltier D Toubai T Henig I. Microbial Metabolite Sensor GPR43 Controls Severity of Experimental GVHD. Nat Commun (2018) 9(1):3674. 10.1038/s41467-018-06048-w 30201970 PMC6131147

[110] Peled JU Devlin SM Staffas A Lumish M Khanin R Littmann ER. Intestinal Microbiota and Relapse After Hematopoietic-Cell Transplantation. J Clin Oncol (2017) 35(15):1650–9. 10.1200/JCO.2016.70.3348 PMC5455763 28296584

